# Immune Checkpoint Inhibitors in Advanced Acral Melanoma: A Systematic Review

**DOI:** 10.3389/fonc.2020.602705

**Published:** 2020-12-03

**Authors:** Qingyue Zheng, Jiarui Li, Hanlin Zhang, Yuanzhuo Wang, Shu Zhang

**Affiliations:** ^1^Department of Dermatology, Peking Union Medical College Hospital, Chinese Academy of Medical Sciences and Peking Union Medical College, Beijing, China; ^2^Eight-year MD Program, Peking Union Medical College, Beijing, China; ^3^Department of Medical Oncology, Peking Union Medical College Hospital, Chinese Academy of Medical Sciences and Peking Union Medical College, Beijing, China

**Keywords:** melanoma, immunotherapy, systematic review, ipilimumab, programmed cell death 1 receptor, radiotherapy, combination drug therapy

## Abstract

**Introduction:**

Acral melanoma (AM) has different biological characteristics from cutaneous melanoma. Although systemic therapeutic strategies for advanced AM resemble those for advanced cutaneous melanoma, the evidence of the clinical use of immune checkpoint inhibitors (ICIs) for AM is still inadequate. We aimed to systematically analyze the therapeutic effects and safety profile of ICI treatments in advanced AM.

**Methods:**

This systematic review was conducted in line with a previously registered protocol. Three electronic databases, conference abstracts, clinical trial registers, and reference lists of included articles were searched for eligible studies. The primary outcomes were therapeutic effects, and the secondary outcomes were the safety profiles.

**Results:**

This systematic review included six studies investigating anti-CTLA-4 immunotherapy, 12 studies investigating anti-PD-1 immunotherapy, one study investigating the combination therapy of anti-CTLA-4 and anti-PD-1, and one study investigating anti-PD-1 immunotherapy in combination with radiotherapy. In most studies investigating ipilimumab, the anti-CTLA-4 antibody, the objective response rate ranged from 11.4 to 25%, the median progression-free survival ranged from 2.1 to 6.7 months, and the median overall survival was more than 7.16 months. For studies discussing anti-PD-1 immunotherapy with nivolumab, pembrolizumab, or JS001, the objective response rate ranged from 14 to 42.9%, the median progression-free survival ranged from 3.2 to 9.2 months, and the median overall survival was more than 14 months. The combination therapy of anti-CTLA-4 and anti-PD-1 immunotherapy showed better efficacy with an objective response rate of 42.9% than single-agent therapy. The retrospective study investigating the combination therapy of anti-PD-1 immunotherapy and radiation showed no overall response. Few outcomes regarding safety were reported in the included studies.

**Conclusions:**

ICIs, especially anti-CTLA-4 monoclonal antibodies combined with anti-PD-1 antibodies, are effective systematic treatments in advanced AM. However, there remains a lack of high-level evidence to verify their efficacy and safety and support their clinical application.

## Introduction

Acral melanoma (AM), a relatively uncommon subtype of melanoma, affects palmar, plantar, and subungual surfaces. Although only comprising 2–3% of all melanoma cases, AM tends to be the most common melanoma subtype in Asian, African, and Hispanic patients, who are at lower risk for sun-related melanoma subtypes (1). Compared with other melanoma subtypes, AM is usually diagnosed at a more advanced stage, which has been proved by the study utilizing the Surveillance, Epidemiology and End Reports (SEER) database (2). Nearly two-thirds of AM was diagnosed at stage II or above, while only approximately one-third of cutaneous melanoma was diagnosed at stage II or above. Therefore, most patients have developed distant metastasis when diagnosed with AM, and systemic treatment for advanced AM is of great significance (3).

Unlike cutaneous melanoma, AM is generally not associated with UV-exposure, which partly accounts for its far lower mutational burdens than cutaneous melanoma. An Australian study demonstrated that three of the 35 (9%) acral melanomas were found to be UVR dominant. The three acral melanomas had biological characteristics similar to the cutaneous melanoma, including elevated total mutational burdens and lower levels of structural variations when compared with acral melanomas with a non-UVR signature (4). AM has different oncogenic drivers from the cutaneous melanoma, including fewer BRAF mutations (10–23%), inconstant *KIT* mutation rates (3–29%), *CCND1* and *CDK4* amplification, and deletion or mutations in different genes, such as *CDK2NA*, *PTEN*, *NF1*, and hTERT (2, 5). However, systemic treatment for advanced AM resembles those for advanced cutaneous melanoma, possibly on account of the limited number of clinical trials evaluating optimal interventions in AM. The responses of AM patients to BRAF-inhibitors are modest as AM has lower frequencies of BRAF mutations (6). AM had different kinds of mutations of KIT, such as copy number gains and activating mutations (7), but targeted therapies with inhibitors such as imatinib usually exert poor or non-durable responses (8). There still remains an urgent need for effective systemic treatment for advanced AM.

Recently, immune checkpoint inhibitors (ICIs) have been recommended as first-line treatment for advanced cutaneous melanoma (9). However, given the low incidence of AM worldwide, few clinical trials reported the therapeutic effects and safety profile of ICIs on the AM. To identify whether ICIs are beneficial for the patients of AM, we conducted this systematic review to analyze the therapeutic effects and safety profile of ICIs in advanced AM.

## Materials and Methods

This systematic review was conducted in line with the protocol registered online in the PROSPERO on May 1, 2020 (ID: CRD42020183476) and was designed in line with the PRISMA guidelines (10).

### Literature Search

Considering the rarity of AM worldwide, we identified all randomized controlled trials (RCTs), prospective observational studies, retrospective studies, and expanded access programs of advanced AM treated with ICIs. Single case reports and narrative reviews were not included. Only the articles published in English or Chinese were included.

Three electronic databases: PubMed, Cochrane Central Register of Controlled Trials (CENTRAL), and EMBASE were searched to identify possibly related studies (from January 1, 1990 to July 20, 2020). Besides, clinical trial registers, conference abstracts, and reference lists of the included studies were also checked for additional possibly relevant studies. The search strategies were shown in the **Supplementary Material**.

### Data Collection and Analysis

In the screening progress, two authors (ZQ and LJ) independently screened the titles and abstracts of the articles identified from the three electronic databases. The articles considered to be potentially relevant would come to the next step, assessing the eligibility. Two authors (ZQ and LJ) assessed the articles according to their full texts. An additional author (ZS) was consulted and resolved possible disagreements. One author (ZH) searched the clinical trial registers, conference abstracts and references of the included studies, and then assessed the eligibility of the records. The included studies must report the response of the patients with unresectable, metastatic, advanced or stage III or IV AM. Two authors (ZQ and LJ) extracted data independently, and a third author (ZS) reviewed the extracted data and made the decision through discussion whenever discrepancies arose. One author (ZQ) used quality assessment tool for before-after (pre-post) studies with no control group, described by the National Heart, Lung, and Blood Institute (NHLBI) (https://www.nhlbi.nih.gov/health-topics/study-quality-assessment-tools), to evaluate the methodological quality of the included studies and the risk of bias.

The primary and secondary outcome data were extracted. The objective response rate (ORR) counted from the sum of complete response (CR) and partial response (PR), median progression-free survival (PFS), median overall survival (OS), the incidence of one-year progression-free survival and the incidence of one-year overall survival were extracted as the primary outcomes to demonstrate the efficacy of the ICIs. As for the safety of ICIs, immune-related adverse event (irAE) rate of all grades and irAE rate of grade 3 or more were extracted as the secondary outcomes. The irAEs were graded in line with the Common Terminology Criteria for Adverse Events (CTCAE).

## Results

**Figure 1:**
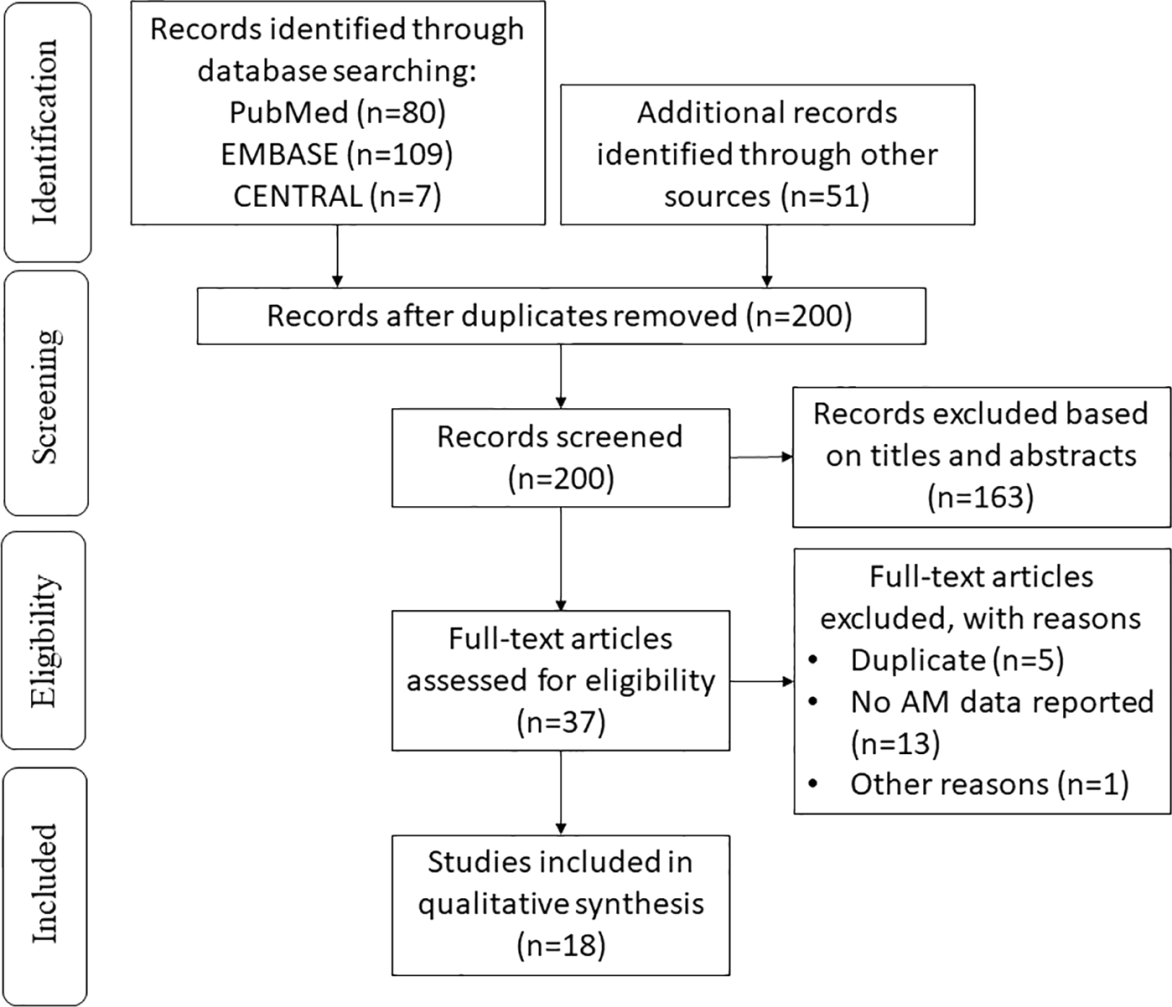
PRISMA 2009 flow diagram of the literature search.

We initially identified 247 records in the literature search process. After removing duplicates, 200 of them remained. After screening, 37 potentially relevant studies were selected, and the full texts were obtained for eligibility assessment. Finally, the primary and secondary outcomes of the 18 records meeting the eligibility criteria were extracted and systemically analyzed (**Figure 1**). The extracted data from the included studies were listed in **Table 1**.

**Table 1:** Characteristics of the 18 studies included in the qualitative review.

Study characteristics								Primary outcomes							Secondary outcomes		Methodological quality
First author and year	Registration ID	Study design	Population	Location	Intervention (mg/kg)	Line of immunotherapy	Record type	ORR	PR	CR	PFS (median)	OS (median)	1-year PFS	1-year OS	All grades irAEs	Grade 3+ irAEs	
Yamazaki 2020 (11)	NCT02717364	prospective, non-interventional, multi-center, observational study	n = 547 (total), n = 107 (<x>ALM</x>)	Japan	ipilimumab(3)	1+	journal article	NR	NR	NR	NR	7.16 months (95% CI, 4.99–10.32 months)	NR	NR	NR	NR	good
Shaw 2012 (12)	NA	EAP	n = 27 (total), n = 5 (AM)	UK	ipilimumab(3)	2+	conference abstract	NR	2 (40%)	NR	NR	NR	NR	NR	NR	NR	poor
Johnson 2015 (13)	NA	retrospective uncontrolled	n = 35 (AM only)	America	ipilimumab(3 or 10)	NR	journal article	11.40%	3 (8.6%)	1 (2.9%)	2.5 months (95% CI, 2.3–2.7 months)	16.7 months (95% CI, 10.9–22.5 months)	NR	NR	20 (57%)	6 (17%)	good
Saberian 2020 (14)	NA	retrospective uncontrolled	n = 44 (AM only)	America	ipilimumab or pembrolizumab or nivolumab	1	conference abstract	17.8% (anti-CTLA-4, n = 17), 40% (anti-PD-1, n = 15)	NR	NR	6.7 months (95% CI, 2.8–17.2 months, anti-CTLA-4), 9.2 months (95% CI, 2.7–19.7 months, anti-PD-1)	38.7 months (95% CI, 7.8–61.6 months, anti-CTLA-4), 60.1 months (95% CI, 12.4–67.4 months, anti-PD-1)	NR	NR	NR	NR	fair
Hafliger 2018 (15)	NA	retrospective uncontrolled	n = 8 (ALM only)	Switzerland	ipilimumab	1	journal article	25%	NR	NR	2.1 months	21 months	NR	NR	NR	NR	fair
Zaremba 2019 (16)	NA	retrospective uncontrolled	n = 21 (AM)	German	anti-PD-1 and anti-CTLA-4 checkpoint inhibitor, respectively	1	journal article	NR	NR	NR	NR	98 months (anti-PD-1, n=16), 95 months (anti-CTLA-4, n=5)	NR	NR	NR	NR	fair
Nathan 2019 (17)	NCT02156804	open-label, single-arm, multi-center phase II study	n = 1,008 (total), n = 55 (AM)	Europe	nivolumab(3)	2+	journal article	NR	NR	NR	NR	25.8 months (95% CI, 15.1-30.6 months)	NR	35 (63.64%)	42 (76.4%)	14 (25.5%)	fair
Yamazaki 2019 (18)	JapicCTI-142533	open-label, single-arm, multicenter phase II study	n = 23 (total), n = 7 (ALM)	Japan	nivolumab(3)	1	journal article	28.6% (90% CI, 10.0-59.1%)	NR	NR	NR	NR	NR	5 (71.4%)	NR	NR	fair
Maeda 2019 (19)	NA	retrospective uncontrolled	n = 68 (total), n = 16 (ALM)	Japan	nivolumab	NR	research letter	19%	3	0	197 days	421 days	NR	NR	NR	NR	fair
Si 2019 (20)	NCT02821000	open-label, non-randomized, multicenter, phase Ib study	n = 102 (total), n = 38 (AM)	China	Pembrolizumab(2)	2	journal article	15.8% (95% CI, 6.0–31.3%)	6 (15.8%)	0	NR	NR	NR	NR	NR	NR	fair
Tang 2019 (21)	NCT02836795	single-center, phase 1, open-label, 2-part (part A dose-escalation and part B dose-expansion) study	n = 36 (total), n = 13 (AM)	China	JS001(1 or 3 or10)	2+	journal article	23%	2	1	NR	NR	NR	NR	NR	0	good
Tang 2020 (22)	NCT03013101	multi-center, single arm, open-label phase II registration study	n = 128 (total), n = 50 (AM)	China	JS001(3)	2+	journal article	14.00%	NR	NR	3.2 months (95% CI, 1.8–3.6 months)	16.9 months (95% CI, 10.9–not estimable months)	5 (10%)	28 (56%)	NR	NR	good
Nakamura 2020 (23)	NA	retrospective uncontrolled	n = 193 (AM only)	Japan	anti-PD-1 antibody	1+	conference abstract	16.60%	13.50%	3.10%	NR	18.1 months	NR	NR	NR	27 (14.0%)	fair
Betof 2020 (24)	NA	retrospective uncontrolled	n = 396 (total), n = 50 (AM)	America	pembrolizumab or nivolumab	NR	journal article	NR	NR	6 (12%)	NR	NR	NR	NR	NR	NR	good
Shoushtari 2016 (25)	NA	multi-institutional, retrospective cohort analysis	n = 60 (total), n = 25 (AM)	America	nivolumab(0.3 to 10) or pembrolizumab(2 or 10)	1+	journal article	32% (95% CI, 15–54%)	6 (24%)	2 (8%)	4.1 months	31.7 months	5 (20%)	5 (20%)	NR	NR	good
Zhao 2019 (26)	NA	retrospective uncontrolled	n = 51 (total), n = 16 (AM)	China	nivolumab(3) or pembrolizumab(2)	1+	journal article	18.75%	3	0	5.3 months (95% CI, 2.4–8.2 months)	NR	NR	NR	NR	NR	fair
Namikawa 2018 (27)	JapicCTI-152869	open-label, single-arm, multi-center phase II study	n = 30 (total), n = 7 (AM)	Japan	nivolumab(1) and ipilimumab(3)	1	journal article	42.9% (95% CI, 9.9–81.6)	NR	NR	NR	NR	3 (43%)	6 (86%)	NR	NR	good
Kato 2019 (28)	NA	retrospective uncontrolled	n = 10 (total), n = 3 (AM)	Japan	radiotherapy and nivolumab(3 or 2) or pembrolizumab(2)	NR	journal article	0	0	0	NR	NR	NR	NR	NR	0	fair

NA, Not Applicable; NR, Not Reported.

### Anti-CTLA-4 Immunotherapy

In the field of anti-CTLA-4 monotherapy, six studies with 177 AM patients treated with ipilimumab were identified (**Table 1**) (11–16). The ORRs for ipilimumab monotherapy ranged from 11.4 to 25%, the median PFS ranged from 2.1 to 6.7 months, and the median OS was more than 7.16 months, demonstrating the therapeutic effects of anti-CTLA-4 immunotherapy in AM. The only study investigating the safety profile of anti-CTLA-4 immunotherapy in AM showed that the frequency of irAEs was 57%, and the frequency of grade 3 or above irAEs was 17%. There remains an unmet need for randomized controlled trials evaluating the anti-CTLA-4 antibodies in AM.

In a prospective, non-interventional, non-controlled, multi-center (146 institutions), observational study, 107 Japanese patients with radically unresectable acral lentiginous melanoma (ALM) receiving ipilimumab had a median OS of 7.16 months (95% CI, 4.99–10.32 months) (11), which was significantly lower than that in other included studies. One possible reason is that the other studies reporting OS all investigated anti-CTLA-4 antibodies as first-line therapy, but this prospective study involved different lines of treatment, in which the patients’ overall health condition was worse. In the results of a published expanded access program, five patients with unresectable stage III/IV AM received 3 mg/kg ipilimumab for up to four cycles. None of them was untreated, and two (40%) patients had a PR (12). A retrospective review of 35 AM patients receiving ipilimumab either 3 mg/kg or 10 mg/kg was conducted in America. One patient achieved CR (2.9%), three achieved PR (8.6%), and four achieved stable disease (SD) (11.4%). The ORR was 11.4%, and the clinical benefit rate (CR + PR + SD) was 22.9%. Of note is that all patients with positive responses were in the 3 mg/kg ipilimumab group. The median PFS was 2.5 months (95% CI, 2.3–2.7months). The median OS was 16.7 months (95% CI, 10.9–22.5 months). In this study, 20 patients (57%) had irAEs of any grade, and 17% patients had grade 3 or 4 events, including colitis (n = 2), hypophysitis (n = 2), hepatotoxicity (n = 1), and skin toxicity (n = 1). No patients died of irAEs (13). In a retrospective analysis of 17 patients with metastatic AM treated with ipilimumab as first-line therapy, the ORR was 17.8%. The median PFS was 6.7 months (95% CI, 2.8–17.2 months), and the median OS was 38.7 months (95% CI, 7.8–61.6 months) (14). A single-center retrospective cohort study conducted in Switzerland involved 8 advanced ALM patients with ipilimumab as the first-line treatment. The ORR was 25%. The median PFS and median OS were 2.1 months and 21 months, respectively (15). A retrospective study conducted in Germany evaluated the therapeutic effects of anti-CTLA-4 and anti-PD-1/PDL1 checkpoint inhibitors, respectively. The five AM patients receiving anti-CTLA-4 monoclonal antibodies as first-line therapy had an OS of 95 months, which was significantly higher in comparison with BRAF inhibitors, MEK inhibitors, and chemotherapy in this study (16).

### Anti-PD-1 Immunotherapy

In the field of anti-PD-1 monotherapy, 12 studies with 494 AM patients treated with anti-PD-1 monoclonal antibodies were identified (**Table 1**). The extracted statistics demonstrated that immunotherapy targeting the interaction between PD-L1 and PD-1 had nearly the same effect as the antibodies targeting CTLA-4 in AM. The ORR ranged from 14 to 40.0%, the median PFS ranged from 3.2 to 9.2 months, and the median OS was more than 421 days in these studies. The only two studies assessing the safety profile of the anti-PD-1 monotherapy in AM patients showed that the rate of grade 3 or above irAEs was between 14.0 and 25.5%. One patient died of grade 5 myasthenia gravis, which should not be neglected. IrAEs should be taken into serious consideration in clinical practice. As the two studies exploring the safety of the anti-PD-1 monotherapy in AM patients involved 193 and 55 AM patients, respectively, the results were relatively convincing (17, 29). These outcomes demonstrated that anti-PD-1 monotherapy could extend the lifespan with tolerable toxicities in part of the patients with advanced AM. However, some patients might encounter serious adverse events, such as grade 3 or above irAEs leading to discontinuation of the therapy and even death.

Three studies assessed nivolumab monotherapy (17–19). In an open-label, single-arm, multi-centered phase II study in Europe (CheckMate 172), 55 patients with unresectable AM and disease progression or recurrence after prior treatment including anti-cytotoxic T-lymphocyte antigen 4 (CTLA-4) monoclonal antibodies received nivolumab intravenously 3 mg/kg every 2 weeks for up to 2 years until progressive disease or intolerable adverse events was observed. The median OS was 25.8 months (95% CI, 15.1–30.6), which was similar to that of patients with non-acral cutaneous melanoma [25.3 months (95% CI, 20.9–28.9)]. The 1-year OS rate was 63.64%. The rate of treatment-related AEs was 76.4%, and the rate of grade 3 or 4 treatment-related AEs was 25.5% (17). Another open-label, single-arm, multi-centered phase II study conducted in Japan explored the nivolumab as first-line treatment in unresectable stage III/IV or recurrent AM. The patients received nivolumab via intravenous infusion 3 mg/kg every 2 weeks in a 6-week cycle until disease progression or unacceptable toxicity happened. The ORR was 28.6% (90% CI, 10.0–59.1%) for the seven ALM patients participating in this study. The 1-year OS rate was 71.4% (18). In a retrospective uncontrolled study to explore the efficacy of nivolumab monoclonal antibodies in ALM in Japan, the 16 ALM patients receiving nivolumab monotherapy had an ORR of 19%. Three of the ALM patients achieved a partial response, and none of them achieved a complete response. The estimated median OS and PFS were 421 and 197 days, respectively. Of note is that among the 13 ALM patients with visceral metastasis, only one achieved a partial response. In comparison, two of the three ALM patients without visceral metastasis achieved a partial response. This phenomenon indicated that the efficacy of nivolumab monotherapy for AM patients might differ in different subgroups (19).

Pembrolizumab was independently assessed in one study (20). In an open-label, non-randomized, multi-centered phase Ib study in China, 38 AM patients received pembrolizumab 2 mg/kg *via* intravenous infusion on day 1 of each 3-week cycle for up to 35 cycles as second-line therapy until disease progression, the onset of intolerable toxicity, investigator decision to discontinue treatment, or voluntary withdrawal of informed consent. As none of the AM patients achieved CR, and six of them achieved PR, the ORR was only 15.8% (95% CI, 6.0–31.3%).

JS001, also known as toripalimab, was independently assessed in two studies, both of which were conducted in China (21, 22). One was a single-center, phase 1, open-label, 2-part (part A dose-escalation and part B dose-expansion) study. Among 13 AM patients refractory to standard systemic treatment, one confirmed CR, two confirmed PR, and three confirmed SD were achieved, with an ORR of 23.1% and a disease control rate of 46.2%. No grade 3 or above irAEs were observed in the involved AM patients, which indicated that JS001 was well-tolerated in this study (21). The other study is a multi-centered, single-arm, open-label phase II registration study. Fifty previously treated advanced AM patients received JS001 3 mg/kg once every two weeks intravenously until disease progression, intolerable toxicity, or voluntary withdrawal of informed consent. The median OS was 16.9 months (95% CI, 10.9–not estimable months), and the median PFS was 3.2 months (95% CI, 1.8–3.6 months). The 1-year OS rate was 56%, and the 1-year PFS rate was 10% (22).

Six retrospective studies evaluated nivolumab and pembrolizumab together (14, 16, 23–26). A study involving 21 Japanese institutions evaluated the efficacy of anti-PD-1 antibodies in 193 advanced AM patients. The CR was 3.1%, and the PR was 13.5%. As a consequence, the ORR was 16.6%. The median OS was reported to be 18.1 months, and irAEs of grades 3 to 5 occurred in 27 patients (14.0%). One patient (0.5%) died of grade 5 myasthenia gravis (23). A study conducted in America involved 50 patients with unresectable stage III or stage IV AM. Six patients (12%) achieved CR (24). A multi-institutional, retrospective cohort analysis conducted in America involved 25 AM patients. Eight of them received nivolumab 0.3 mg/kg to 10 mg/kg intravenously every 2 to 3 weeks. Seventeen AM patients received pembrolizumab either 2 mg/kg every 3 weeks or 10 mg/kg every 2 to 3 weeks. As two AM patients had a CR, and six had a PR, the ORR was 32% (95% CI, 15–54%). The median PFS was 4.1 months, and the median OS was 31.7 months. The 1-year PFS rate was 20%, and the 1-year OS rate was also 20% (25). A study involving 16 metastatic AM patients was conducted in China. The patients received nivolumab 3 mg/kg every 2 weeks, or received pembrolizumab 2 mg/kg every 3 weeks by intravenous infusion. None of the patients achieved CR, and three patients achieved PR. The median PFS was 5.3 months (95% CI, 2.4–8.2 months) (26). Another study conducted in Germany evaluated the efficacy of anti-PD-1/PDL1 and anti-CTLA-4 monoclonal antibodies, respectively. The 16 AM patients receiving anti-PD-1 antibodies as first-line therapy had an OS of 98 months, which was significantly higher in comparison with BRAF inhibitors, MEK inhibitors, and chemotherapy in this study (16). In an analysis of 15 patients with metastatic AM who received pembrolizumab or nivolumab as the first-line treatment, the ORR was 40%. The median PFS of the 15 patients was 9.2 months (95% CI, 2.7–19.7 months), and the median OS was 60.1 months (95% CI, 12.4–67.4 months) (14).

### Combination Therapy of Anti-CTLA-4 and Anti-PD-1 Monoclonal Antibodies

One study involving seven AM patients assessed combination therapy of ipilimumab and nivolumab (**Table 1**) (27). An open-label, single-arm, multi-centered phase II study conducted in Japan treated patients with confirmed unresectable stage III/IV or recurrent AM with two doses of nivolumab (1 mg/kg) intravenously plus ipilimumab (3 mg/kg) per cycle for two 3-week cycles, then 6-week cycles with biweekly nivolumab (3 mg/kg) as first-line therapy. The ORR was 42.9% (95% CI, 9.9–81.6), and the number of patients with 1-year PFS and 1-year OS was 3 (43%) and 6 (86%), respectively.

### Combination Therapy of Anti-PD-1 Immunotherapy and Radiotherapy

The efficacy and safety of anti-PD-1 immunotherapy and radiotherapy were investigated in one retrospective study conducted in Japan. Three AM patients received one of the following regimens: 3 mg/kg nivolumab every 2 weeks; 2 mg/kg nivolumab every 3 weeks; or 2 mg/kg pembrolizumab every 3 weeks. They were all treated with radiotherapy after the progression of anti-PD-1. None of the patients achieved PR or SD, and two patients achieved SD. There was no grade 3 or above irAEs (28).

## Discussion

This systematic review included 16 studies with 542 advanced AM patients and provided a general overview of the efficacy and safety profile of immune checkpoint inhibitors in advanced AM. We conclude that ICIs generally demonstrated remarkable clinical efficacy and acceptable irAEs for most patients.

### Anti-CTLA-4 Monotherapy and Anti-PD-1 Monotherapy

High-level evidence of the therapeutic effects and safety profile of anti-CTLA-4 and anti-PD-1 monotherapy in AM is still limited, and its therapeutic effects need to be confirmed *via* high-quality randomized controlled trials. There are three uncompleted clinical trials evaluating anti-PD-1 antibodies for AM patients, which involve different kinds of antibodies from different companies, such as IBI308, IBI310, and pembrolizumab. Two of them were randomized controlled trials. The NCT04277663 will study IBI310 combined with IBI308 in comparison to high-dose interferon in AM removed by surgery. The NCT03698019 will study pembrolizumab in stage III or IV high-risk melanoma before and after surgery. With more clinical trials, the therapeutic effects and safety profile of anti-PD-1 monotherapy will be illustrated more clearly.

### Combination Therapy of Anti-CTLA-4 and Anti-PD-1 Immunotherapy

Previous research in cutaneous melanoma showed that the combination of anti-CTLA-4 monoclonal antibodies and anti-PD-1 monoclonal antibodies was more effective but more toxic than single-agent therapy (30, 31). The only study evaluating the therapeutic effects of anti-CTLA-4 (ipilimumab) in combination with anti-PD-1 (nivolumab) in advanced AM showed an ORR of 42.9%, a 1-year PFS rate of 43%, and a 1-year OS rate of 86%, which were all much higher than those of anti-CTLA-4 or anti-PD-1 immunotherapy alone, demonstrating that administering nivolumab plus ipilimumab may provide a more hopeful treatment choice for patients with AM than either agent alone.

However, as the number of patients involved in the study was not enough to exert a convincing conclusion, more clinical trials evaluating the therapeutic effects and safety profile of the combined therapy of anti-CTLA-4 and anti-PD-1 are needed. The NCT02978443 is an uncompleted biomarker study of advanced mucosal melanoma or ALM treated with the combination of ipilimumab and nivolumab.

### Combination Therapy of Anti-PD-1 Immunotherapy and Radiotherapy

Radiotherapy is now seldom used due to the remarkable success of targeted therapy and immunotherapy, as well as melanoma’s low susceptibility to radiotherapy. Nevertheless, several studies discovered that radiation combined with immune checkpoint inhibitors had a synergistic effect in advanced cutaneous melanoma (32, 33). This systematic review included one retrospective study that assessed the anti-PD-1 immunotherapy combined with radiation (28). The ORR was 0, and the rate of grade 3 or above irAEs was also 0. As only three AM patients were involved in this study, the credibility and convincement of this evidence are poor, calling for more relevant studies to solve this problem. In theory, radiotherapy can enhance the transport of T cells to tumor tissues and enhance the strength of specific anti-tumor immune responses (34), so the combination of ICIs and radiotherapy may be more effective than monotherapy.

### Combination Therapy of Tyrosine Kinase Inhibitor and ICIs

As melanomas often overexpress VEGF, which may play a significant role in disease progression, anti-angiogenesis targeting VEGF is a meaningful strategy in treating melanoma (35). Although there is no completed clinical trial investigating the combination of tyrosine kinase inhibitor and ICIs in AM, some clinical trials are recruiting patients, which will fill the gaps in this field. The NCT03955354 investigates the combination of anti-PD-1 monoclonal antibody SHR-1210 and Apatinib as first-line therapy in advanced AM. The NCT03991975 studies the TQB2450, a kind of PD-L1 antibodies, combined with Anlotinib in patients with advanced AM.

### Different Effects of ICIs in AM and Non-Acral Cutaneous Melanoma

Some studies identified in this systematic review compared the therapeutic effects of immune checkpoint inhibitors in AM and other subtypes of melanoma. A retrospective study found that in anti-PD-1 monotherapy, patients with AM (12%) were less likely to have a CR compared to cutaneous melanoma (30.9%) (24). In an open-label, nonrandomized, multi-centered, phase Ib study evaluating the efficacy of pembrolizumab as second-line therapy, the ORR was 15.8% (95% CI, 6.0–31.3%) in AM, 19.5% (95% CI, 8.8–34.9%) in non-acral melanoma (20). An open-label, single-arm, multi-centered phase II study showed that in combination therapy of anti-CTLA-4 and anti-PD-1 immunotherapy, the ORR of patients with AM (42.9%) was much lower than that of patients with non-acral cutaneous melanoma (75.0%) (27). However, a retrospective study found that therapy containing pembrolizumab had the same effect in AM (ORR 26.7%) as in the non-acral cutaneous subtype (ORR 26.7%) (36). Although the quality and size of each one of the studies was not enough to provide strong evidence, the evidence that supports AM has worse efficacy outcomes when treated with ICIs compared with cutaneous melanoma overweighs the few evidence for the same efficacy outcomes. Although the exact reason for the worse efficacy outcomes in AM compared to cutaneous melanoma in most studies was unclear, several studies have revealed unique biological characteristics of AM, which may contribute to uncovering the underlying reason. Unlike cutaneous melanoma, AM is generally not linked to UV-exposure, which results in its far lower mutational burdens than cutaneous melanoma. A study using whole-genome sequencing showed that single-nucleotide variant were 1.02–3.68 per Mb in AM, which is much lower than that in cutaneous melanoma (37). The frequencies of somatic structural variants were more in acral than in cutaneous melanomas, and greater proportions of the acral and mucosal melanoma genomes had copy number variation (38, 39). AM also has different oncogenic drivers from cutaneous melanoma, including inconstant *KIT* mutation rates (3–29%), *CCND1* and *CDK4* amplification, and deletion or mutations in different genes, such as *CDK2NA*, *PTEN*, *NF1*, and hTERT (2). A few studies suggested that the response to immunotherapy is associated with tumor mutational burden, and increased tumor neoantigen load may predict the objective response (40–43). This may partly explain why the efficacy of ICIs for AM is lower than that for the non-acral cutaneous subtype.

A possible reason is that PD-L1 expression is lower in AM than that in the non-acral cutaneous subtype. One study reported the expression of the PD-L1 in different subtypes of melanoma. 33% of AM had PD-L1 expression, compared with 62% of the sun-damaged melanomas (44). As anti-PD-1 antibodies target the interaction between PD-1 and PD-L1, the PD-L1 expression might be a biomarker predictive of the response to ICIs (45, 46).

The tumor microenvironment may also play a role. In a study, grade III TILs were more frequent in cutaneous non-ALM than in ALM (33.3 vs. 22.6%, p = 0.033), and lower TIL levels (p = 0.031) were significantly associated with shorter OS (47). However, in a study from Korea, there was no significant association between nodular melanoma, superficial spreading melanoma, and ALM with respect to the presence of lymphocytes or LS and DFS and OS (48). So whether there is a difference in TIL in the tumor microenvironment between AM and cutaneous melanomas remains to be determined. As the skin in acral sites is strikingly different from the skin in other anatomical sites, including differences of melanocyte differentiation and the absence of hair follicles and sebaceous glands, the differences between the microenvironment of AM and cutaneous melanoma may suggest a different response rate for ICIs.

### Limitations and Prospects

We recognized several limitations in this systematic review. First, the methodological quality of 11 out of 18 studies included in this systematic review was evaluated as poor or fair, and 10 out of 18 studies were retrospective, together with the lack of randomized controlled trials, may result in biases. The number of studies involved in this review was also small due to the limited exploration in this field. Second, the ICIs were applied in mixed lines of therapy in most studies. Nevertheless, ICIs may have variable efficacy and safety outcomes as first-line and further-line treatment of AM. For instance, a prospective study showed that the OS result in treatment-naive AM patients was longer than in those who had received prior treatment when treated with anti-CILA-4 antibodies (11). The conclusion would be more convincing if the studies separated the patients into different subgroups according to the lines of treatment when they received ICIs. Third, most of the studies did not report the primary location of AM, or did not analyze the outcomes of different subgroups of primary sites, but the response to treatment might differ in different primary site of AM. According to a multi-center retrospective study in China, there exist differences in survival in different primary locations in AM. Compared with AM arising from sole, AM arising from palm and nail bed subgroup has a better prognosis (49). AM in different anatomical positions may have variable mutation profiles, which is exemplified by the study result that BRAF mutations were more often found in AM located on the feet. Comparing AM arising from dorsal acral sites with AM on palms and soles, lower frequencies of NRAS (25 *versus* 39.1%) and NF1 (0 *versus* 17.3%) and higher frequencies of BRAF (75 *versus* 21.7%) and TERT promoter (50 *versus* 8.6%) mutations were observed (16). As the variable genetic changes in varying anatomical positions likely influence biological behavior and therapeutic response, it is worthwhile to evaluate the therapeutic effects and safety profile of ICIs in AM arising from specific primary sites. Last, most included studies did not report the outcomes concerning the irAEs of ICIs in AM separately, so the safety of ICIs in AM remains an unsettled question that needs to be further explored.

There remain several directions of exploration in the application of ICIs in the AM. First, the most suitable clinical setting for the ICIs must be defined to achieve satisfactory outcomes. High-quality clinical trials focusing on ICIs in combination with radiotherapy, chemotherapy, or other immunotherapies in the treatment for AM are in urgent need, especially the randomized controlled trials involving statistically sufficient patients. In addition, the appropriate neoadjuvant and adjuvant therapy also needs to be explored, which could not be accomplished without the efforts and contributions of countries including China where AM is one of the most prevalent melanoma subtypes. Second, there lack laboratory models of AM, which hinders the development of new treatments such as ICIs. Third, prognostic biomarkers that can predict the response of AM to ICIs should be further explored. Tumor neoantigen load and PD-L1 expression level are regarded as promising biomarkers, but the reliability of them in AM needs to be verified, as they might not be applied in the actual situation (50). In a retrospective study, the PD-L1 expression level was not associated with anti-PD-1 ORR (p = 0.982) in AM (14). Besides the two markers, lower infiltration of cancer-associated fibroblasts and expression of cancer-associated fibroblast markers are linked to the positive response to anti-PD-1 monoclonal antibodies in AM (51), which is worth further exploring. Finally, possibly effective treatments for AM after the ICI treatment fails also need to be considered. Targeted therapy, or other immunotherapies, even other kinds of ICIs might be effective. In a clinical trial, nivolumab had desirable efficacy and safety results after tumor progression on prior ipilimumab (17), which brought hope to these patients.

## Conclusions

In conclusion, ICIs generally demonstrated remarkable clinical efficacy and acceptable irAEs in patients with advanced AM. ICIs, especially anti-CTLA-4 immunotherapy combined with anti-PD-1 immunotherapy, are promising therapeutic strategy for advanced AM. Nevertheless, there remains a lack of high-level proof to verify their safety and support their clinical application. The effect of ICIs in AM from different primary sites should also be further elucidated in future studies. We hope that this systematic review could benefit physicians and patients, and pave the way for further research on the treatment of advanced AM.

## Author Contributions

QZ and JL conceived and designed this review. QZ, JL, and HZ conducted the literature search and collected the data. QZ drafted the manuscript and figures. SZ, HZ, JL, and YW reviewed and revised the manuscript. All authors contributed to the article and approved the submitted version.

## Conflict of Interest

The authors declare that the research was conducted in the absence of any commercial or financial relationships that could be construed as a potential conflict of interest.
