# Supplementary material for: Immune Checkpoint Inhibitors in Advanced Acral Melanoma: A Systematic Review
Source: Front Oncol. 2020 Dec 3;10:602705. doi: 10.3389/fonc.2020.602705 (PMC7744720; doi:10.3389/fonc.2020.602705)
Supplement: Supplementary file 1 [file Table_1.docx]

**PubMed** (updated: 20 July 2020)

(("melanoma"[Mesh]) OR (melanoma*[Title/Abstract]) OR (malignant melanoma*[Title/Abstract]) OR (melanoma*, malignant[Title/Abstract])) AND ((acral*[Title/Abstract]) OR (acral melanoma[Title/Abstract])) AND (("Antibodies" [MeSH Terms]) OR (immune*[Title/Abstract]) OR (CTLA-4[Title/Abstract]) OR (CTLA4[Title/Abstract]) OR (cytotoxic T-lymphocyte-associated[Title/Abstract]) OR (ipilimumab[Title/Abstract]) OR (yervoy[Title/Abstract]) OR (programmed cell death protein[Title/Abstract]) OR (PD-1[Title/Abstract]) OR (PDCD1[Title/Abstract]) OR (pembrolizumab[Title/Abstract]) OR (keytruda[Title/Abstract]) OR (nivolumab[Title/Abstract]) OR (opdivo[Title/Abstract]) OR (toripalimab [Title/Abstract]) OR (JS001[Title/Abstract]) OR (cemiplimab[Title/Abstract]) OR (libtayo[Title/Abstract]) OR (PD-L1[Title/Abstract]) OR (CD274[Title/Abstract]) OR (atezolizumab[Title/Abstract]) OR (tecentriq[Title/Abstract]) OR (durvalumab[Title/Abstract]) OR (imfinzi[Title/Abstract]) OR (avelumab[Title/Abstract]) OR (bavencio[Title/Abstract]))

**Embase** (updated: 20 July 2020)

#1 'acral melanoma'/exp

#2 'acral melanoma*':ab,kw,ti

#3 'acral neoplasm*':ab,kw,ti

#4 #1 or #2 or #3

#5 'antibody'/exp

#6 'immune*':ab,kw,ti

#7 CTLA-4:ab,kw,ti

#8 CTLA4:ab,kw,ti

#9 'cytotoxic T-lymphocyte-associated':ab,kw,ti

#10 ipilimumab:ab,kw,ti

#11 yervoy:ab,kw,ti

#12 'programmed cell death protein':ab,kw,ti

#13 PD-1:ab,kw,ti

#14 PDCD1:ab,kw,ti

#15 pembrolizumab:ab,kw,ti

#16 keytruda:ab,kw,ti

#17 nivolumab:ab,kw,ti

#18 opdivo:ab,kw,ti

#19 toripalimab:ab,kw,ti

#20 JS001:ab,kw,ti

#21 cemiplimab:ab,kw,ti

#22 libtayo:ab,kw,ti

#23 PD-L1:ab,kw,ti

#24 CD274:ab,kw,ti

#25 atezolizumab:ab,kw,ti

#26 tecentriq:ab,kw,ti

#27 durvalumab:ab,kw,ti

#28 imfinzi:ab,kw,ti

#29 avelumab:ab,kw,ti

#30 bavencio:ab,kw,ti

#31 #5 OR #6 OR #7 OR #8 OR #9 OR #10 OR #11 OR #12 OR #13 OR #14 OR #15 OR #16 OR #17 OR #18 OR #19 OR #20 OR #21 OR #22 OR #23 OR #24 OR #25 OR #26 OR #27 OR #28 OR #29 OR #30

#32 #4 AND #31

**Cochrane library** (updated: 20 July 2020)

#1 MeSH descriptor: [Melanoma] explode all trees

#2 Melanoma*:ti,ab,kw (Word variations have been searched)

#3 Malignant Melanoma*:ti,ab,kw (Word variations have been searched)

#4 #1 OR #2 OR #3

#5 acral melanoma*:ti,ab,kw (Word variations have been searched)

#6 acral neoplasm*:ti,ab,kw (Word variations have been searched)

#7 acral*:ti,ab,kw (Word variations have been searched)

#8 #5 OR #6

#9 (#4 AND #7) OR #8

#10 MeSH descriptor: [Antibodies] explode all trees

#11 immune*:ti,ab,kw (Word variations have been searched)

#12 CTLA-4:ti,ab,kw (Word variations have been searched)

#13 CTLA4:ti,ab,kw (Word variations have been searched)

#14 cytotoxic T-lymphocyte-associated:ti,ab,kw (Word variations have been searched)

#15 ipilimumab:ti,ab,kw (Word variations have been searched)

#16 yervoy:ti,ab,kw (Word variations have been searched)

#17 programmed cell death protein:ti,ab,kw (Word variations have been searched)

#18 PD1:ti,ab,kw (Word variations have been searched)

#19 PD-1:ti,ab,kw (Word variations have been searched)

#20 PDCD1:ti,ab,kw (Word variations have been searched)

#21 pembrolizumab:ti,ab,kw (Word variations have been searched)

#22 keytruda:ti,ab,kw (Word variations have been searched)

#23 nivolumab:ti,ab,kw (Word variations have been searched)

#24 opdivo:ti,ab,kw (Word variations have been searched)

#25 toripalimab:ti,ab,kw (Word variations have been searched)

#26 JS001:ti,ab,kw (Word variations have been searched)

#27 cemiplimab:ti,ab,kw (Word variations have been searched)

#28 libtayo:ti,ab,kw (Word variations have been searched)

#29 PD-L1:ti,ab,kw (Word variations have been searched)

#30 CD274:ti,ab,kw (Word variations have been searched)

#31 atezolizumab:ti,ab,kw (Word variations have been searched)

#32 tecentriq:ti,ab,kw (Word variations have been searched)

#33 durvalumab:ti,ab,kw (Word variations have been searched)

#34 imfinzi:ti,ab,kw (Word variations have been searched)

#35 avelumab:ti,ab,kw (Word variations have been searched)

#36 bavencio:ti,ab,kw (Word variations have been searched)

#37 #10 OR #11 OR #12 OR #13 OR #14 OR #15 OR #16 OR #17 OR #18 OR #19 OR #20 OR #21 OR #22 OR #23 OR #24 OR #25 OR #26 OR #27 OR #28 OR #29 OR #30 OR #31 OR #32 OR #33 OR #34 OR #35 OR #36

#38 #9 AND #37

**Trial register** (updated: 20 July 2020)

Search term: acral melanoma

US National Institutes of Health Ongoing Trials Register (www.clinicaltrials.gov)

**Conference abstracts** (January 2014- July 2020)

Search term: acral melanoma

American Society of Clinical Oncology (ASCO)

European Society of Medical Oncology (ESMO)
